# Antibody Response to Toscana Virus and Sandfly Fever Sicilian Virus in Cats Naturally Exposed to Phlebotomine Sand Fly Bites in Portugal

**DOI:** 10.3390/microorganisms7090339

**Published:** 2019-09-11

**Authors:** André Pereira, Nazli Ayhan, José Manuel Cristóvão, Hugo Vilhena, Ângela Martins, Patrícia Cachola, Joaquim Henriques, Mónica Coimbra, Ana Catarino, Tereza Lestinova, Tatiana Spitzova, Petr Volf, Lenea Campino, Remi Charrel, Carla Maia

**Affiliations:** 1 Global Health and Tropical Medicine (GHMT), Instituto de Higiene e Medicina Tropical (IHMT), Universidade Nova de Lisboa (UNL), 1349-008 Lisboa, Portugal; andrepereira.vet@gmail.com (A.P.); jcristovao@ihmt.unl.pt (J.M.C.); 2 Medical Parasitology Unit, IHMT-UNL, 1349-008 Lisboa, Portugal; lcampino@ihmt.unl.pt; 3 Unité des Virus Emergents (UVE: Aix Marseille Univ, IRD 190, INSERM 1207, IHU Méditerranée Infection), 13385 Marseille, France; nazliayhann@gmail.com (N.A.); remi.charrel@univ-amu.fr (R.C.); 4 Center for Investigation Vasco da Gama (CIVG), Department of Veterinary Medicine, Vasco da Gama Universitary School, 3020-210 Coimbra, Portugal; hcrvilhena@hotmail.com; 5 University Veterinary Hospital of Coimbra, 3020-210 Coimbra, Portugal; 6 Animal and Veterinary Research Centre (CECAV), University of Trás-os-Montes and Alto Douro, 5000-801 Vila Real, Portugal; 7 Hospital Veterinário da Arrábida, 2925-538 Azeitão, Portugal; vetarrabida.lda@gmail.com; 8 Hospital Veterinário do Algarve, 8000-072 Faro, Portugal; clinicavetcentral@sapo.pt; 9 Hospital Veterinário de Berna, 1050-039 Lisboa, Portugal; oncovet@gmail.com; 10 Clínica Veterinária Porto Seguro, 8700-507 Olhão, Portugal; cvetps@gmail.com; 11 VetCoa - Serviços Veterinários, 6320-354 Sabugal, Portugal; anacata75@gmail.com; 12 Department of Parasitology, Faculty of Science, Charles University, 128 00 Prague, Czech Republic; terka.kratochvilova@seznam.cz (T.L.); tatiana.spitzova@gmail.com (T.S.); volf@cesnet.cz (P.V.)

**Keywords:** arbovirus, *Bunyavirales*, cat, *Phenuiviridae*, *Phlebotomus perniciosus*, *Phlebovirus*, saliva, Sandfly Fever Sicilian Virus, Toscana virus

## Abstract

Phlebotomine sand fly-borne pathogens such as *Leishmania* spp. and phleboviruses are emerging threats to humans and animals worldwide. The aim of this work was to evaluate the exposure of cats from Portugal to Toscana virus (TOSV) and Sandfly Fever Sicilian virus (SFSV) and assess the associated risk factors. The possible association between exposure to *Phlebotomus perniciosus* saliva with TOSV and SFSV was also investigated. Out of 369 cats tested, 18 (4.9%, *n* = 365) were seropositive for TOSV, and eight (2.2%, *n* = 367) were seropositive for SFSV. Multivariate logistic regression analysis showed that cats presenting clinical signs that were compatible with leishmaniosis and antibodies to TOSV had a significantly higher risk of being SFSV seropositive. The presence of antibodies to sand fly-borne viruses in cats indicate that these animals are frequently exposed to sand flies and transmitted pathogens. Data suggest that cats can be used to qualitatively monitor human exposure to TOSV and SFSV in endemic areas. The clinical impact of SFSV in cats’ health should be investigated. The identification of the sand fly species responsible for the circulation of TOSV and SFSV in nature and the evaluation of the vectorial competence of *P. perniciosus* to SFSV should also be addressed.

## 1. Introduction

Phlebotomine sand fly-borne agents such as protozoa of the *Leishmania* genus and phleboviruses are emerging threats to humans and animals worldwide [1]. Viruses belonging to the *Phlebovirus* genus (family *Phenuiviridae*) that are pathogenic to humans such as Toscana virus (TOSV) and Sandfly Fever Sicilian virus (SFSV) are endemic in the Mediterranean region [2]. Most human infections are asymptomatic or mild influenza-like syndromes that remain undocumented; however, outbreaks of acute meningitis or meningoencephalitis due to TOSV have been reported in most of the countries bordering the Mediterranean Sea [3]. Recent studies indicate that humans and domestic animals from European countries are heavily exposed to TOSV and SFSV, with reported seroprevalence rates up to 50% for TOSV and up to 9.2% for SFSV in humans [4], and up to 15% for TOSV and 85% of SFSV in dogs [5]. In Portugal, human cases of TOSV meningitis [6] and one case of prolonged febrile illness due to SFSV [7] have been reported. The presence of antibodies to SFSV ranging from 0.2% to 4.3% and to TOSV ranging from 2% to 5.3% have also been demonstrated in seroprevalence studies conducted in humans [6,8,9]. In addition, seroprevalence rates up to 6.8% and 3.7% for TOSV and up to 50.8% and 1.6% for SFSV have been reported in dogs and cats, respectively [10]. Portugal is endemic also for human and canine leishmanioses, and the number of feline leishmaniosis and *L. infantum* infection reports in cats has increased [11]. Several sand fly species of the *Larroussius* subgenus are vectors of *L. infantum*, of which *Phlebotomus perniciosus* is the main vector in the west part of Mediterranean [12]. Apart from *P. perniciosus*, other species from this subgenus are also proven or putative vectors of TOSV and SFSV [3,13]. When the sand fly female takes a blood meal, it injects saliva into the vertebrate host, evoking the development of a specific antibody response in the host. The use of antibody response to sand fly saliva has been proposed as a biomarker to estimate the exposure of dogs and humans living in endemic areas of leishmaniosis to sand flies [14]. Nevertheless, no data are available regarding the possible use of sand fly saliva as a biomarker of exposure to phleboviruses. Thus, the aim of the present study was to evaluate the exposure of cats to TOSV and SFSV and associated risk factors, and to investigate a possible association between exposure to *P. perniciosus* saliva with TOSV and SFSV.

## 2. Materials and Methods

### 2.1. Animals and Samples

From April to December 2017, a total of 369 cats with access to outdoors from veterinary medical centres, animal shelters and colonies from four Portuguese continental NUTS (Nomenclature of Units for Territorial Statistics) II, were studied (Table 1). Peripheral blood (1–2 mL) was obtained by cephalic or jugular venipuncture from each animal and collected into EDTA and serum-separating tubes. Serum, plasma, and buffy coat were obtained from each animal by centrifugation and stored at −20 °C until used in serological analyses (serum and plasma) and DNA extraction (buffy coat). Whenever available, individual data were recorded for each cat. Of these, 350 serum and buffy coat samples had been previously used in an epidemiological study regarding exposure to *P. perniciosus* saliva and the presence of *Leishmania* infection [15]. Antibodies to sand fly saliva were detected in 167 (47.7%) cats, while *Leishmania* infection was detected in 26 (7.7%) cats [15]. The procedures were approved by the Ethical committee (authorization no. 8 2011-PI) of Instituto de Higiene e Medicina Tropical and for the Portuguese veterinary authorities (authorization no. 008637/18-04-2019) as complying with the Portuguese legislation for the protection of animals (Decree-Law nº 113/2013). Consent was obtained from the owner of the cat or the person in charge of the rescue associations for stray cats.

### 2.2. Virus Microneutralization Assay

Each plasma was tested using microneutralization assay as previously described [10], with minor modifications. Briefly, twofold serial dilutions of 15 μL of plasma samples were mixed with an equal volume of 100 TCID_50_ of TOSV or SFSV into 96-well plates, providing twofold final dilutions between 1:20 and 1:160. The cut-off value for positivity was set at titre ≥40 [16].

### 2.3. Detection of Leishmania Infection

Anti-*Leishmania* antibodies were determined by IFAT (immunofluorescence antibody test; cut-off value at a titre ≥64) in the 16 sera samples that were previously screened [15]. Briefly, an *L. infantum* MON-1 (MCAN/PT/05/IMT-373) suspension of 10^7^ promastigotes was used as antigen, and the anti-cat IgG (whole molecule)-FITC was used in a dilution of 1:20. A serum sample from a seropositive cat (IFAT titre 1204) was used as positive control, while the serum sample of a cat from a non-endemic country of leishmaniosis was used as negative control [17]. The detection of *Leishmania* DNA in the buffy coat was done using a nested PCR protocol with genus-specific primers targeting the *SSU*-rDNA gene [18]. A positive control containing *L. infantum* MON-1 (MHOM/PT/88/IMT-318) DNA and a negative control without DNA template were included in each amplification.

DNA amplicons were resolved by electrophoresis on 1.5% agarose gels stained with Green Safe Premium (Nzytech, Portugal), using a 100-bp DNA ladder as a molecular weight marker, and then visualized under UV illumination. Cats were considered infected by *Leishmania* if they tested positive for at least one of the techniques.

### 2.4. Statistical Analysis

Confidence intervals (95% CI) for proportions were obtained by the Wilson method. To avoid confounding, a multivariate logistic regression analysis was performed to identify the putative risk factors associated with the presence of antibodies against SFSV/TOSV in cats. A first exploratory data analysis using simple logistic regression models (univariate analysis) was conducted to select a set of variables with a *P*-value ≤ 0.20 for the Wald test. Subsequently, a backward stepwise elimination procedure was implemented, using a *P*-value ≤ 0.05 for the Wald test as the criterion for variables to remain in the multivariate model. Multicollinearity assessment was produced by linear regression analysis with tolerance and variance inflation factor (VIF) options. Finally, the likelihood ratio test (*G^2^*), the Hosmer and Lemeshow goodness-of-fit test, and the determination of the area under the receiver operating characteristic curve (AUC) were performed to evaluate the model validity. All statistical analyses were conducted using IBM® SPSS® Statistics version 25.0 and OpenEpi version 3.01 software.

## 3. Results

Eighteen (4.9%, *n* = 365) and eight (2.2%, *n* = 367) of the tested cats had neutralizing antibodies at a titre ≥40 for TOSV and SFSV, respectively (Table 1). Three cats had antibodies reacting against both viruses. Multivariate logistic regression analysis showed that the independent variables clinical signs (β = 1.924; χ^2^_Wald_ = 4.756, df = 1; *P* = 0.029) and antibodies to TOSV (β = 2.630; χ^2^_Wald_ = 8.174; df = 1; *P* = 0.04) were significant predictors of SFSV seropositivity. Additionally, none of them presented tolerance and VIF values less than 0.2 and exceeding 10, respectively, therefore revealing the absence of potential collinearity problems [19]. According to the fitted model (*p* = $\frac{1}{{1+e}^{-\left[-6.126\;+\;1.804\;\mathrm{Clinical}\;\mathrm{Signs}\;\left(\mathrm{Yes}\right)\;+2.360\;\mathrm{TOSV}\;\mathrm{antibodies}\;\left(\mathrm{Yes}\right)\right]}}$), the probability of a cat being SFSV seropositive is higher if it presents clinical signs compatible with leishmaniosis (e.g., muscular atrophy, dermatological or ocular manifestations, lameness, lymphadenopathy, lethargy, pale mucous membranes and weight loss) (adjusted OR = 6.8; 95% CI = 1.2–38.6) and antibodies to TOSV (adjusted OR = 13.9; 95% CI = 2.3–84.2). This model was fit to the data well (Hosmer and Lemeshow test = 2.896, df = 2, *P* = 0.235) and showed an excellent discriminatory ability (AUC = 0.809; *P* = 0.003) [19].

On the other hand, none of the independent variables studied presented a significant predictor effect on the presence of antibodies to TOSV in cats, as revealed by the likelihood ratio test (*G^2^* = 3.644, df = 1, *P* = 0.056).

## 4. Discussion

The detection of neutralizing antibodies against TOSV and SFSV, together with the presence of antibodies to *P. perniciosus* saliva, indicates that cats are frequently exposed to sand flies and consequently to sand fly-transmitted pathogens.

The seroprevalence to both phleboviruses observed here was in the same order of magnitude as those previously reported in cats from the south of Portugal (TOSV: 3.7%; SFSV: 1.6%) [10], reinforcing that these animals can harbor both phleboviruses. Although the rate of neutralizing antibodies against TOSV and SFSV in humans cannot be inferred from the rate observed in domestic animals, as the rates can be 10 times higher in animals than in humans (and vice versa)*,* our data suggest that cats be used for qualitatively assessing human exposure to TOSV and SFSV in endemic areas. In addition, as both phleboviruses cause disease in humans, the role of cats as reservoirs or amplifying hosts for these pathogens should be investigated. Further, and since results point out to a wide dispersion of sand fly-borne phleboviruses in Portugal, serological studies at the national level should be performed on humans to assess their level of exposure. Awareness of physicians to include these viruses in the differential diagnosis in patients presenting unexplained febrile illness or neuroinvasive infections should also be raised. 

Although the capacity of SFSV to cause disease in cats is currently unknown, an association between the presence of clinical signs compatible with leishmaniosis and the exposure to this phlebovirus was observed; whether the clinical signs were related to SFSV rather to *Leishmania* infection remain uncertain, it cannot be ruled out that both pathogens can cause similar clinical manifestations. Nevertheless, and from an animal health point of view, veterinarians should include SFSV in the differential diagnosis of cats suspected of having a vector-borne disease.

The detection of different sand fly-borne phleboviruses in sand flies [20], humans [9], or dogs [21] sharing the same environmental area has been reported in Portugal. Therefore, the detection of neutralizing antibodies to both phleboviruses in three cats, or the significantly higher risk of cats presenting antibodies to TOSV to be SFSV seropositive was not surprising and confirms the co-circulation of both viruses in the same region. 

An age-dependent increase in both SFSV and TOSV-specific immunity was observed in the tested cats, which was probably related to a cumulative exposure of older animals to the vectors, although the difference was not statistically significant. Similar trends were noticed between SFSV positivity and *P. perniciosus* saliva and between anti-TOSV seropositivity and the non-use of ectoparasiticides, lifestyle, and region without statistical significance; however, a non-equal sample size by variable could lead to stochastic fluctuations of seroprevalence rates. Additionally, data suggest the cats sharing the same environment with other animals and having short fur length are more exposed to TOSV and SFSV, which can be explained by the ecology and anatomy of vector sand fly species, despite the association between variables not being statistically significant. Nevertheless, the lack of statistical association between phlebotomine activity period and TOSV antibody positivity can be explained by the long-lasting antibody response against TOSV, which can remain months after virus exposure [22].

The epidemiological relationship between human [23] and canine [24] leishmaniosis and TOSV infection have previously been suggested. However, in the present study, no association between antibodies to *Leishmania* and/or parasite DNA with TOSV or SFSV positivity was detected. Further studies to evaluate if *Phlebovirus* infection favours *Leishmania* infection and vice versa should be conducted.

Despite human TOSV and SFSV cases in humans and domestic animals have been reported in Portugal, along with the detection of antibodies to both viruses, up to now, none of them has been detected or isolated from sand flies. Therefore, the identification of the sand fly species responsible for their circulation in nature is needed. Given the low detection in nature of co-infections by different pathogens in phlebotomine sand flies [25,26], the mechanism leading to a double serological reactivity in vertebrate hosts is more likely due to successive bites by sand flies, of the same or of different species, infected by a single pathogen [23,24]. Five sand fly species are endemic in Portugal, namely: *P. ariasi*, *P. papatasi*, *P. perniciosus*, *P. sergenti*, and *Sergentomyia minuta* [27]. Despite SFSV having historically been associated with *P. papatasi*, this sand fly species is unlikely to be the responsible for its circulation in Portugal, as it is present in low densities, and has a restricted geographical distribution. *P. perniciosus* is a proven vector of TOSV [28,29,30,31,32] and is the most abundant *Phlebotomus* spp. present in the areas where cats were surveyed [12,33]. As recent studies have suggested that SFSV and SFSV-like viruses may be transmitted by alternative vectors belonging to *Larroussius* subgenus [3,13], together with the detection in the present study of IgG against *P. perniciosus* saliva in cats seropositive to SFSV, the vectorial competence of this sand fly species to SFSV should be investigated.

## 5. Conclusions

The presence of antibodies to both TOSV and SFSV in cats, together with the previous detection of both pathogens in humans and dogs, indicate the exposure to and co-circulation of sand fly-borne phleboviruses in Portugal. Our results reinforce the need to increase public and animal health awareness regarding phleboviruses and to improve diagnostic tools for the rapid pathogen detection and identification of both sand flies and vertebrate hosts.

## Figures and Tables

**Table 1:** Seroprevalence of antibodies against Sandfly Fever Sicilian virus (SFSV) and Toscana virus (TOSV) in cats from Portugal.

Variable/Categories	Antibodies to SFSV		Antibodies to TOSV	
	Tested, *n* (%)	Positive, *n* (%; CI)	Tested, *n* (%)	Positive, *n* (%; CI)
Sex	366		364	
Female	204 (55.7)	2 (1.0; 0.3–3.5)	203 (55.8)	10 (4.9; 2.7–8.8)
Male	162 (44.3)	6 (3.7; 1.7–7.8)	161 (44.2)	8 (5.0; 2.5–9.5)
Age group	330		328	
2–11 months	76 (23.0)	0 (0.0; 0.0–4.8)	77 (23.5)	1 (1.3; 0.2–7.0)
12–35 months	71 (21.5)	1 (1.4; 0.2–7.6)	71 (21.6)	3 (4.2; 1.4–11.7)
36–95 months	91 (27.6)	2 (2.2; 0.6–7.6)	91 (27.7)	4 (4.4; 1.7–10.7)
More than 95 months	92 (27.9)	5 (5.4; 2.3–12.1)	89 (27.1)	8 (9.0; 4.6–16.8)
Reproductive status	352		350	
Entire	239 (67.9)	4 (1.7; 0.7–4.2)	240 (68.6)	9 (3.8; 2.0–7.0)
Neutered	113 (32.1)	3 (2.7; 0.9–7.5)	110 (31.4)	9 (8.2; 4.4–14.8)
Breed	366		364	
Defined	14 (4.6)	0 (0.0; 0.0–21.5)	16 (4.4)	2 (12.5; 3.5–36.0)
Mongrel	349 (95.4)	8 (2.3; 1.2–4.5)	348 (95.6)	16 (4.6; 2.9–7.3)
Fur length	366		364	
Short	328 (89.6)	8 (2.4; 1.2–4.7)	327 (89.8)	17 (5.2; 3.2–8.2)
Medium or long	38 (10.4)	0 (0.0; 0.0–9.2)	37 (10.2)	1 (2.7; 0.5–13.8)
Lifestyle	362		360	
Domestic	152 (42.0)	4 (2.6; 1.0–6.6)	148 (41.1)	13 (8.8; 5.2–14.5)
Shelter/stray	210 (58.0)	3 (1.4; 0.5–4.1)	212 (58.9)	4 (1.9; 0.7–4.8)
Region	367		365	
Center	62 (16.9)	2 (3.2; 0.9–11.0)	60 (16.4)	8 (13.3; 6.9–24.2)
Lisbon metropolitan area	285 (77.7)	6 (2.1; 1.0–4.5)	285 (78.1)	8 (2.8; 1.4–5.4)
Alentejo	5 (1.4)	0 (0.0; 0.0–43.5)	5 (1.4)	1 (20.0; 3.6–62.4)
Algarve	15 (4.1)	0 (0.0; 0.0–20.4)	15 (4.1)	1 (6.7; 1.2–29.8)
Other animals	362		360	
No	45 (12.4)	0 (0.0; 0.0–7.9)	43 (11.9)	3 (7.0; 2.4–18.6)
Yes	317 (87.6)	7 (2.2; 1.1–4.5)	317 (88.1)	13 (4.1; 2.4–6.9)
Ectoparasiticides	350		348	
No	277 (79.1)	4 (1.4; 0.6–3.7)	275 (79.0)	9 (3.3; 1.7–6.1)
Yes	73 (20.9)	4 (5.5; 2.2–13.3)	73 (21.0)	8 (11.0; 5.7–20.2)
Clinical signs	367		365	
No	258 (70.3)	2 (0.8; 0.2–2.8)	258 (70.7)	12 (4.7; 2.7–8.0)
Yes	109 (29.7)	6 (5.5; 2.5–11.5)	107 (29.3)	6 (5.6; 2.6–11.7)
Concomitant diseases	187		185	
No	97 (51.9)	2 (2.1; 0.6–7.2)	97 (52.4)	4 (4.1; 1.6–10.1)
Yes	90 (48.1)	4 (4.4; 1.7–10.9)	88 (47.6)	9 (10.2; 5.4–18.3)
Phlebotomine activity period	367		365	
No	201 (54.8)	3 (1.5; 0.5–4.3)	200 (54.8)	7 (3.5; 1.7–7.0)
Yes	166 (45.2)	5 (3.0; 1.3–6.9)	165 (45.2)	11 (6.7; 3.8–11.5)
Antibodies to *Phlebotomus perniciosus* saliva	326		325	
Negative	178 (54.6)	1 (0.6; 0.1–3.1)	178 (54.8)	5 (2.8; 1.2–6.4)
Positive	148 (45.4)	7 (4.7; 2.3–9.4)	147 (45.2)	10 (6.8; 3.7–12.1)
Antibodies to *Leishmania* and/or parasite DNA	367		365	
Negative	344 (93.7)	8 (2.3; 1.2–4.5)	342 (93.7)	17 (5.0; 3.1–7.8)
Positive	23 (6.3)	0 (0.0; 0.0–14.3)	23 (6.3)	1 (4.3; 0.8–21.0)
Antibodies to TOSV	363		NA	
Negative	345 (95.0)	5 (1.4; 0.6–3.3)		
Positive	18 (5.0)	3 (16.7; 5.8–39.2)		
Antibodies to SFSV	NA		363	
Negative			355 (97.8)	15 (4.2; 2.6–6.8)
Positive			8 (2.2)	3 (37.5; 13.7–69.4)
Total	367	8 (2.2; 1.1–4.2)	365	18 (4.9; 3.1–7.7)

Abbreviations: CI, 95% confidence interval; NA, not applicable.
